# QTL mapping of agronomic traits in tef [*Eragrostis tef *(Zucc) Trotter]

**DOI:** 10.1186/1471-2229-7-30

**Published:** 2007-06-12

**Authors:** Ju-Kyung Yu, Elizabeth Graznak, Flavio Breseghello, Hailu Tefera, Mark E Sorrells

**Affiliations:** 1 Department of Plant Breeding and Genetics, Cornell University, Ithaca NY 14853, USA; 2 Debre Zeit Agricultural Research Center, P.O. Box 32, Debre Zeit, Ethiopia; 3 Syngenta Seeds Inc. 317 330th Street, Stanton, MN 55018, USA; 4 Embrapa Arroze Feijão, Caixa Postal 179, Santo Antônio de Goiás, GO 75375-000, Brazil

## Abstract

**Background:**

Tef [*Eragrostis tef *(Zucc.) Trotter] is the major cereal crop in Ethiopia. Tef is an allotetraploid with a base chromosome number of 10 (2n = 4× = 40) and a genome size of 730 Mbp. The goal of this study was to identify agronomically important quantitative trait loci (QTL) using recombinant inbred lines (RIL) derived from an inter-specific cross between *E. tef *and *E. pilosa *(30-5).

**Results:**

Twenty-two yield-related and morphological traits were assessed across eight different locations in Ethiopia during the growing seasons of 1999 and 2000. Using composite interval mapping and a linkage map incorporating 192 loci, 99 QTLs were identified on 15 of the 21 linkage groups for 19 traits. Twelve QTLs on nine linkage groups were identified for grain yield. Clusters of more than five QTLs for various traits were identified on seven linkage groups. The largest cluster (10 QTLs) was identified on linkage group 8; eight of these QTLs were for yield or yield components, suggesting linkage or pleotrophic effects of loci. There were 15 two-way interactions of loci to detect potential epistasis identified and 75% of the interactions were derived from yield and shoot biomass. Thirty-one percent of the QTLs were observed in multiple environments; two yield QTLs were consistent across all agro-ecology zones. For 29.3% of the QTLs, the alleles from *E. pilosa *(30-5) had a beneficial effect.

**Conclusion:**

The extensive QTL data generated for tef in this study will provide a basis for initiating molecular breeding to improve agronomic traits in this staple food crop for the people of Ethiopia.

## Background

Tef, *Eragrostis tef *(Zucc.) Trotter, is a major food grain in Ethiopia but is a minor cereal crop worldwide. The primary use of tef is for grinding into flour to make injera, a spongy fermented flat bread that is a staple food for most Ethiopians. The vegetative portions of the plant are also an important source of fodder for livestock. In Ethiopia for the crop year 2003–2004, it occupied two million hectares, which represented 28% of the area grown with eight cereal crops in the country [1]. The ability of tef to perform well on both waterlogged Vertisols in the highlands as well as water-stressed areas in the semi-arid regions throughout the country is one of the reasons for which tef is preferred over other grain crops such as maize or barley [2]. In addition, tef generally suffers less from biotic stresses compared to most other cereal crops grown in Ethiopia and it contains high levels of proteins and mineral [3].

Tef is an allotetraploid species with a base chromosome number of 10 (2n = 4× = 40). It belongs to the family Poaceae, sub-family *Eragrostidae *and genus *Eragrostis*. The genus contains approximately 350 species [4]. The exact diploid progenitors of tef are still unknown; however, most researchers agree that *E. pilosa *is the species most closely related to *E. tef *and is considered the direct wild tetraploid progenitor of tef [5]. It is also the only species known to be cross-compatible with modern tef varieties. Flow cytometry research has shown that tef has a genome size of 730 Mbp [6], which is roughly the same size as diploid sorghum and about 60% larger than the diploid rice genome. It has also the smallest chromosomes reported among the *Poaceae *ranging from 0.8 to 2.9 μm [6], which has significantly hindered the cytogenetic research of this species.

Understanding the genetic control of agronomic traits is essential for the sustained improvement of tef. Lodging is the number one cause of yield loss in tef; even with good crop management practices. Recent studies in tef have shown strong correlations between lodging, panicle type, culm thickness, and grain yield [2,7]. Important agronomic traits in tef, as in most crop species, are quantitative inherited [7,8], which complicates genetic analysis. Quantitative trait locus (QTL) analysis allows the identification of discrete chromosome segments controlling complex traits [9]. The significance of identifying QTLs that correspond with certain traits is that the information can be used for marker-assisted selection (MAS) program. This is the most comprehensive report of QTL analyses for agronomic traits in tef to date.

Cultivated tef and the wild species, *E. pilosa*, differ greatly for most agronomic traits and the close relationship betweenthese two species facilitate hybridization providing a unique opportunity to develop a new pool of genetic variation. The study by Tefera et al. [7] has demonstrated that *E. pilosa *has contributed useful breeding traits, such as earliness and short stature. Therefore, utilization of *E. pilosa *as a donor in an inter-specific cross is a useful strategy for broadening the genetic diversity of the existing gene pool in cultivated tef.

The purpose of this research was to identify and characterize QTLs controlling 22 agronomic traits; eight yield-related traits and 14 morphological traits, in the inter-specific cross between *E tef*, cv. Kaye Murri and *E. pilosa *(30-5).

## Results

### Trait analysis

Effects of years and locations were highly significant (*p *< 0.001) for all traits evaluated in multiple locations (data not shown). The variance among lines was highly significant (*p *< 0.001) for all traits except RPR1, RPR2, and Crush1 (data not shown). The mean value of the two parents, Kaye Murri and *E. pilosa *(30-5) were significantly different for all 22 traits (Table 1). As expected for an inter-specific cross, distribution of phenotypic values in the progeny showed bi-directional transgressive segregants for all traits, except Crush1 and Crush2, which showed transgressive segregants towards the *E. pilosa *(30-5) parent only.

**Table 1:** Traits, phenotypes of RIL population, parents (*E. tef *cv. Kaye Murri, KM and *E. pilosa *(30-5), Ep), and evaluation environments.

			**RIL**				**Parent**			
						
**Trait**	**Abbv.**	**Unit**	**Mean**	**Min**	**Max**	**SD**	**KM**	**Ep**	**Norm.**	**Experiments**
**Yield and Yield Related Traits**										
Heading date***	HD	days	30.50	21.25	47.50	7.13	44.00	32.50	log	e09,10,11
Marturity date***	MD	days	81.75	65.25	107.00	11.18	96.00	84.25		e09,10,11
Panicle weight***	PWt	g	0.32	0.06	1.16	0.16	0.71	0.23	log	e01,02,03,04,05,06,07,08,09,10,11
Panicle seed weight***	PSWt	g	0.18	0.01	0.66	0.11	0.47	0.12	log	e01,02,03,04,05,06,07,08,09,10,11
100 seed weight***	100sw	mg	17.25	6.50	32.50	4.23	26.75	17.75		e01,02,03,04,05,07,08
Grain yield***	GY	g	156	7.25	707.50	130.23	319	165	sqrt	e01,02,03,04,05,06,07,08,09,10,11
Shoot biomass***	SB	g	986	196	4050	755	1650	750	sqrt	e01,02,03,04,05,06,07,08,09,10,11
Lodging index***	Lodg	score	71.00	35.00	99.50	14.61	65.13	81.50		e01,02,03,04,05,07,08,09,10,11
										
**Morphological and Plant Height Related Triats**										
Culm length***	CulmL	cm	44.50	22.02	71.55	9.32	56.40	42.25		e01,02,03,04,05,06,07,08,09,10,11
Culm diameter1^a^***	CD1	cm	1.30	0.72	2.10	0.23	1.72	1.04		e01,02,03,04,05,06,07
Culm diameter2^b^***	CD2	cm	1.29	0.66	2.08	0.24	1.76	1.11		e01,02,03,04,05,06,07
Peduncle length***	PedL	cm	19.35	9.75	29.65	3.63	19.93	17.80		e01,02,03,04,05,06,07,08,09,10,11
Panicle length***	PanL	cm	23.75	12.50	39.75	4.35	30.90	20.95		e01,02,03,04,05,06,07,08,09,10,11
Plant height***	PH	cm	71.45	37.80	99.95	12.30	88.23	61.70		e01,02,03,04,05,06,07
Number of internodes***	Ninter	score	3.00	2.25	4.45	0.40	3.35	2.88		e01,02,03,04,05,06,07
1st internode length***	Inter1	cm	6.65	2.70	13.75	1.62	8.40	6.20		e01,02,03,04,05,06,07,08,09,10,11
2nd internode length***	Inter2	cm	10.45	5.40	16.95	2.20	12.88	9.83		e01,02,03,04,05,06,07,08,09,10,11
Crown diameter***	Dia	cm	1.55	0.83	2.23	0.28	2.08	1.14		e01,03,07
Rind penetrometer1^c^	RPR1	lbs	0.54	0.28	0.83	0.11	1.15	0.45		e04
Rind penetrometer2^d^	RPR2	lbs	0.36	0.24	0.65	0.08	0.74	0.30		e04
Crush strength1^e^	Crush1	lbs	4.88	1.98	6.88	1.05	9.49	3.59		e04
Crush strength2^f^***	Crush2	lbs	4.06	1.17	7.67	1.11	9.64	3.24		e04

Abbr. = abbreviation of trait; Norm. = transformation used to achieve normality. Eight locations representing three agro-ecologies in Ethiopia; Akaki (AK), Alemtena (AL), Debre Zeit Black Soil (DZBS), Debre Zeit Light Soil (DZLS), Denbi (DE), Melkasa (MEL), Chefe (CH) and Holetta (HO), wet semi-arid in higher than 1900 masl altitude (C2-1; AK, CH, HO), wet semi-arid in 1700–1900 masl altitude (C2-2; DZBS, DZLS, DE), dry semi-arid in lower than 1700 masl altitude (C3-3; AL, MEL). Each experiment representing the combination of different environments and years for each trait evaluation; AK and 2000 (e01), AL and 2000 (e02), DZBS and 2000 (e03), DZLS and 2000 (e04), DE and 2000 (e05), MEL and 2000 (e06), CH and 2000 (e07), HO and 2000 (e08), AK and 1999 (e09), AL and 1999 (e10) and DZBS and 1999 (e11).

^a ^culm diameter of 1^st ^internode

^b ^culm diameter of 2^nd ^internode

^c ^measurement of penetration strength in 1^st ^internode rind

^d ^measurement of penetration strength in 2^nd ^internode rind

^e ^measurement of crushing strength in 1^st ^internode

^f ^measurement of crushing strength in 2^nd ^internode

*** The analysis of variance for traits among lines and experiments at significance of 0.001 probability level

Phenotypic correlations were estimated between the overall means of the 22 phenotypic traits. All traits, except RPR1 and RPR2, were highly correlated (*p *< 0.001) with at least one other trait. Significant positive correlations were identified between yield and most agronomic traits except PedL and Dia in this population (Table 2). Lodging was not correlated with traits supposedly lodging related, such as PH, RPR1, 2 and Crush1, 2 (Table 2). The frequency distributions of most of traits fit the normal distribution, however, seven traits (PWt, PSWt, GY, SB, HD, RPR1 and RPR2) were significantly skewed, and transformation was applied prior to QTL analysis except RPR1 and 2. The traits, RPR1, RPR2 and Crush1 were excluded for QTL analyses which did not show variances among lines thus, 19 traits were evaluated for QTL analyses.

**Table 2:** Trait correlations for grain yield and lodging index.

	**GY**	**Lodg**
**HD**	0.50***	0.14
**MD**	0.48***	-0.06
**PWt**	0.67***	0.21*
**PSWt**	0.75***	0.28**
**100sw**	0.50***	0.41***
**GY**		0.51***
**SB**	0.87***	0.37***
**Lodg**	0.51***	
**CulmL**	0.60***	0.25*
**CD1**	0.42***	-0.06
**CD2**	0.42***	-0.06
**PedL**	-0.19	-0.28**
**PanL**	0.52***	0.07
**PH**	0.58***	0.16
**Ninter**	0.53***	0.23*
**Inter1**	0.41***	0.15
**Inter2**	0.46***	0.23*
**Dia**	0.16	-0.22*
**RPR1**	0.05	-0.13
**RPR2**	0.04	-0.13
**Crush1**	0.14	-0.09
**Crush2**	0.45***	-0.02

*, ** and *** significant at the 0.05, 0.01 and 0.001 probability level, respectively.

**Figure 1:**
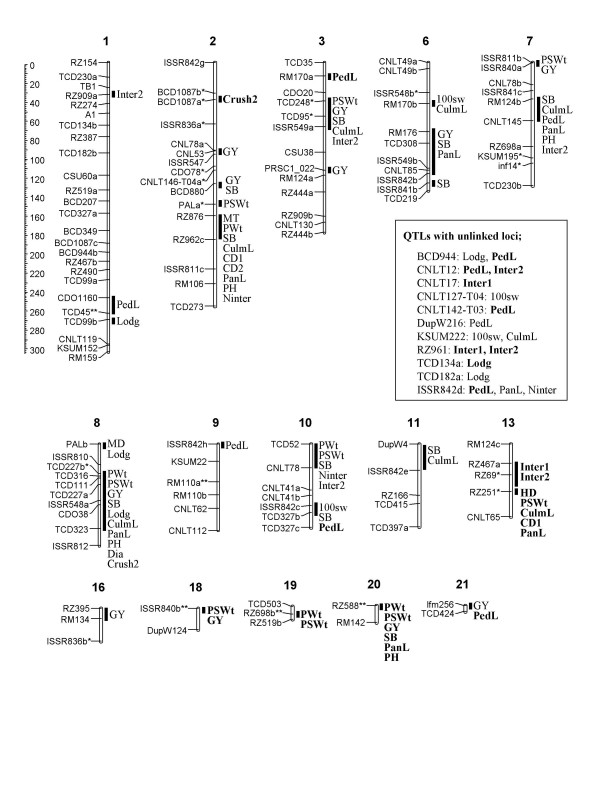
**Molecular linkage map with positions of QTLs for 19 traits on tef RIL population; *E. tef *× *E. pilosa *(30-5)**. The genetic distance in centimorgans (cM) is given on the left at the top. Six linkage groups are not presented because they did not contain significant QTLs. QTLs with the increasing effect contributed by *E. pilosa *(30-5) are in boldface.

A total of 99 QTLs for 19 traits was identified by three analyses in common; SMR, CIM and MT-CIM. The map positions of the QTLs together with the additive effects and *R*^2 ^values from CIM are presented in Fig. 1 and Table 3. The QTLs were distributed over all linkage groups except 4, 5, 12, 14, 15, and 17 (Fig. 1). Two or more QTLs were identified for all traits except HD, CD2 and Dia. The number of chromosomes with significant QTL for the specific traits ranged from one (HD, CD2 and Dia) to 12 (GY). The number of significant QTL for the specific chromosomes ranged from zero (LG4, 5, 12, 14, 15, and 17) to 14 (LG2) (Fig. 1). The wild relative, *E. pilosa *(30-5) alleles had an increasing effect on 29.3% of the QTLs in the present study.

**Table 3:** QTLs detected by composite interval mapping in the RIL population from the cross '*E. tef *× *E. pilosa (30-5)*'

**Trait^a^**	**Chrom.**	**Closest locus/loci^b^**	**Peak^c^**	**LOD**	**R2**	**Add^d^**	**Exp.^e^**
**HD**	13	RZ251		4.47	0.16	0.01	e11
							
**MD**	2	RZ876 ~ RZ962c	RZ876	3.08	0.34	-1.70	e09, e10
	8	PALb		3.42	0.20	-3.14	e11
							
**PWt**	2	RZ876 ~ RZ962c	RZ876	3.18	0.15	-0.06	e05
	8	ISSR548a		3.97	0.15	-0.06	e06
	10	TCD52		3.51	0.19	-0.08	e04
	19	RZ698b		3.25	0.14	0.07	e01
	20	RZ588		3.30	0.23	0.09	e07
							
**PSWt**	2	PALa		4.41	0.17	-0.10	e04
	3	TCD95		3.80	0.21	-0.10	e09
	7	ISSR811b ~ ISSR840a	ISSR811b	5.20	0.24	-0.12	e06, e10
	8	ISSR548a		3.35	0.13	-0.07	e06
	10	TCD52		3.99	0.27	-0.12	e04
	13	RZ251		3.02	0.11	0.10	e08
	18	ISSR840b		5.45	0.24	0.13	e04, e8
	19	RZ698b		3.17	0.14	0.09	e01
	20	RZ588		3.51	0.20	0.11	e07
							
**100sw**	6	RM170b		5.21	0.26	-2.22	e02, e03
	10	ISSR842c ~ TCD327b	ISSR842c	6.23	0.21	-1.90	e03, e07
	un	CNLT127-T04		5.25	0.21	-1.75	e05
	un	KSUM222		5.78	0.30	-2.49	e01
							
**GY**	2	CNL53 ~ ISSR547	CNL53	4.06	0.11	-1.13	e04, e06, e08
	2	BCD880		5.84	0.24	-1.47	e02
	3	TCD248 ~ TCD95	TCD95	6.34	0.28	-1.07	e03, e05, e07, e09, e10, e11
	3	PRSC1_022		3.98	0.12	-1.23	e01
	6	TCD308 ~ ISSR842b	ISSR549b	6.36	0.20	-1.61	e08, e09
	7	ISSR840a		5.94	0.25	-1.67	e01, e06, e10
	8	TCD227a ~ ISSR548a	ISSR548a	4.88	0.15	-1.02	e04, e11
	8	TCD323		4.92	0.15	-1.12	e02
	16	RZ395 ~ RM134	RZ395	3.35	0.12	-0.59	e05, e09
	18	ISSR840b		3.48	0.12	0.97	e07
	20	RZ588		3.98	0.18	1.10	e06, e11
	21	lfm256		5.03	0.14	-1.29	e05, e03
							
**SB**	2	BCD880		3.94	0.14	-2.33	e05
	2	RZ962c		3.04	0.15	-2.40	e04
	3	TCD248 ~ ISSR549a	ISSR549a	6.57	0.19	-1.63	e01, e02, e09, e10, e11
	6	RM176 ~ ISSR549b	RM176	4.72	0.16	-2.39	e04, e05, e06, e08, e09
	6	ISSR841b		3.13	0.11	-1.90	e03
	7	CNLT145		4.60	0.26	-2.88	e03
	8	ISSR548a ~ TCD323	ISSR548a	6.60	0.21	-2.28	e06, e11
	10	TCD52 ~ CNLT78		6.32	0.26	-0.90	e07, e09
	10	TCD327b		3.07	0.12	-1.97	e03
	11	DupW4 ~ ISSR842e	DupW4	3.28	0.15	-1.42	e10, e11
	20	RZ588		4.36	0.22	1.71	e10
							
**Lodg**	1	TCD99b		3.87	0.25	-6.12	e03
	8	PALb		5.50	0.38	-7.12	e02
	8	TCD323		5.53	0.23	-6.00	e05
	un	BCD944a		5.68	0.32	-7.21	e05
	un	TCD182a		4.90	0.23	-5.60	e02
							
**CulmL**	2	RZ876		3.67	0.23	-2.18	e02
	3	TCD95		5.92	0.21	-2.28	e09, e10
	6	RM170b		3.34	0.12	-1.92	e08
	7	RM124b ~ CNLT145	CNLT145	3.44	0.34	-2.64	e02
	8	ISSR548a		3.46	0.12	-1.49	e11
	11	DupW4 ~ ISSR842e	ISSR842e	5.66	0.28	-2.16	e07, e09, e11
	13	RZ251		3.32	0.24	2.20	e07, e08
	un	KSUM222		3.57	0.22	-2.50	e01
							
**CD1**	2	RZ876 ~ RZ962c	RZ876	4.67	0.33	-0.12	e05
	13	RZ251		3.39	0.21	0.09	e04
							
**CD2**	2	RZ876 ~ RZ962c	RZ876	4.86	0.33	-0.12	e05
							
**PedL**	1	CDO1160 ~ TCD45	CDO1160	4.23	0.19	-0.86	e07
	3	RM170a		5.90	0.25	1.40	e08
	7	CNLT145		3.17	0.11	-0.94	e08
	9	ISSR842h		3.43	0.12	-0.68	e01
	10	ISSR842c		5.35	0.21	1.08	e10
	21	lfm256		3.72	0.11	0.78	e02, e04, e11
	un	BCD944a		4.11	0.17	1.06	e04
	un	CNLT12		4.05	0.17	0.98	e02, e03
	un	DupW216		3.32	0.35	-1.12	e09
	un	ISSR842d		3.16	0.17	1.00	e11
	un	CNLT142-T03		3.50	0.13	0.72	e01
							
**PanL**	2	RZ876		3.36	0.11	-1.27	e03
	6	RM176		3.52	0.10	-1.22	e03, e06
	7	RM124b		3.92	0.14	-1.07	e10
	8	ISSR548a ~ CD038	ISSR548a 4.73	0.14	-1.46	e03	
	13	RZ251		4.38	0.19	1.78	e05
	20	RZ588		4.00	0.22	1.36	e07
	un	ISSR842d		3.25	0.12	-0.98	e10
							
**PH**	2	RZ876 ~ RZ962c	RZ962c 3.63	0.26	-3.79	e01, e05	
	7	CNLT145		3.14	0.13	-3.26	e04
	8	ISSR548a		4.35	0.14	-2.91	e03
	20	RZ588		3.82	0.15	2.52	e07
							
**Ninter**	2	RZ876 ~ RZ962c	RZ962c 4.97	0.20	-0.11	e01, e02, e05	
	10	TCD52		4.61	0.16	-0.13	e04
	un	ISSR842d		3.51	0.17	-0.11	e01
							
**Inter1**	13	RZ69 ~ RZ251	RZ69	4.20	0.22	0.62	e02
	un	CNLT17		4.61	0.24	0.58	e02
	un	CNLT142-T03		3.95	0.18	-0.30	e03, e07
	un	RZ961		3.66	0.33	0.84	e04, e08
							
**Inter2**	1	RZ909a		4.30	0.14	-0.35	e07
	3	TCD95 ~ ISSR549a	TCD95	4.72	0.21	-0.75	e06, e07
	7	CNLT145		3.02	0.14	-0.57	e08
	10	CNLT78		3.31	0.15	-0.51	e01
	13	RZ467a ~ RZ69	RZ69	3.80	0.26	0.78	e01, e02
	un	CNLT12		4.46	0.34	0.71	e02, e03
	un	RZ961		3.53	0.16	0.38	e07
							
**Dia**	8	ISSR548a		3.11	0.15	-0.07	e03
							
**Crush2**	2	BCD1087a		3.08	0.14	0.49	
	8	ISSR548a		5.31	0.17	-0.48	

^a ^See Methods, designations of each trait

^b ^Flanking markers within the significance threshold at each border of the QTL range in the most significant experiments

^c ^Peak marker is the marker closest to the peak LOD score if QTL covered more than two loci.

^d ^Positive value of additive effect (Add) means the increased effect for the QTL was caused by the *E. pilosa (30-5) *allele

^e ^See the legend of Table 1, designations of each experiment

A test for potential interactions between significant QTL marker loci for all traits identified a relatively small number of epistatic interactions between loci. A total of 20 interactions consisting of 18 marker loci for four traits were identified across nine linkage groups and three unlinked loci (Table 4).

**Table 4:** Significant two-way interactions between marker loci determined using Epistat program.

**Trait**	**Marker1**		**Marker2**		**MC-test**
			
	**Name**	**Chr.**	**Name**	**Chr.**	
GY	CNL53	2	TCD227a	8	0.0028
GY	lfm256	21	CNLT85	6	0.013
GY	lfm256	21	RZ588	20	0.0031
GY	TCD95	3	lfm256	21	0.0168
GY	TCD95	3	TCD227a	8	0.0007
SB	ISSR549a	3	CNLT78	10	0.0024
SB	ISSR549a	3	ISSR841b	6	0.0019
SB	ISSR549a	3	ISSR842e	11	0.0013
SB	RM176	6	CNLT145	7	0.0016
SB	RM176	6	ISSR549a	3	0.0005
SB	RM176	6	ISSR549b	3	0.0027
SB	RM176	6	ISSR842e	11	0.0001
SB	RM176	6	RZ588	20	0.0046
SB	RZ962c	2	ISSR842e	11	0.0001
SB	TCD95	3	CDO38	8	0.0046
PedL	CNLT12	un	BCD944a	un	0.0047
PedL	CNLT12	un	CNLT145	7	0.0003
PanL	RM176	6	RZ588	20	0.0069
Inter2	CNLT145	7	RZ961	un	0.0021
Inter2	CNLT145	7	ISSR549a	3	0.0011

* Monte Carlo simulation to evaluate significance of interaction

### QTL for grain yield and yield related traits

#### Heading date (HD) and maturity date (MD)

Two MD QTLs were identified at three locations representative of all three agro-ecologies. The MD QTL on LG2 at 24.8 cM explained 0.34 of *R*^2^, and was associated with yield related traits such as PWt and SB (Fig. 1). Early maturity is a common characteristic of wild relatives of tef and *E. pilosa *(30-5) matured on average 12 days earlier than Kaye Murri. On the other hand, at the QTL for HD, the allele from *E. pilosa *contributed longer cycle.

#### Panicle weight (PWt)

Five QTLs were identified for PWt on LG2, 8, 10, 19 and 20 and *R*^2 ^ranged from 14% to 23%. The QTL interval on LG2 (RZ876 to RZ962c), was associated with two yield related traits and six morphological traits. All five QTLs were overlapped or closely located with the QTLs for PSWt. Three of the QTLs were positively affected by Kaye Murri resulting in weight increase.

#### Panicle seed weight (PSWt)

Nine QTLs were identified for PSWt covering all three agro-ecologies with six locations. Out of seven QTLs that were associated with GY, five Kaye Murri QTLs showed a positive effect. Four PSWt QTLs were associated with PWt and two overlapped with GY QTLs. However, there was no QTL associated with 100sw.

#### 100 seed weight (100sw)

Four QTLs were identified for 100sw, all of which were increased by the alleles of the cultivated parent. No 100sw QTL were associated with PWt, PSWt or GY QTL.

#### Grain yield (GY)

The largest number of QTLs was identified for GY, among the traits studied. Twelve QTLs were identified in nine linkage groups. The highest LOD score was 6.39 for ISSR549b explaining 0.2 of *R*^2^. Two QTLs in LG3, 50 cM apart, were significant in six locations representing three agro-ecologies. The *E. pilosa (30-5) *alleles in LG18 (ISSR840b) and LG20 (RZ588) increased grain yield. The rest of the QTLs were positively affected by the Kaye Murri alleles.

#### Shoot biomass (SB)

The most significant QTLs for SB were found on LG3, 8 and 10 with a LOD > 6 and *R*^2 ^> 0.19. One QTL on LG20 (RZ588) explained 0.22 of *R*^2 ^and the positive allele was from *E. pilosa (30-5)*. This QTL co-located with PWt, PSWt and GY QTLs, all with same positive alleles from *E. pilosa (30-5)*.

#### Lodging index (Lodg)

Three QTLs were located on LG1 and 8, and two QTLs were associated with unlinked loci. All five QTL alleles contributed by Kaye Murri increased lodging. The two QTLs (PALb and TCD323) on LG8 were located in the distal region of the linkage group. PALb showed the highest *R*^2 ^(0.38) and highest LOD score (5.5) and co-segregated with MD. TCD323 co-located with SB and GY, and was located near eight other QTLs, including lodging related traits, such as Crush2.

### QTL for morphological and plant height related traits

#### Culm length (CulmL)

Eight significant CulmL QTLs were identified on seven linkage groups and one unlinked locus (Table 3). The *R*^2 ^ranged from 0.12 to 0.34. Except for RZ251 on LG13, increasing effects of all significant QTLs came from Kaye Murri. The strongest CulmL QTL is TCD95 on LG3 with a LOD score of 5.92 and an *R*^2 ^value of 0.21. This locus was associated with PSWt, Inter2, GY and SB.

#### Culm diameter 1^st ^and 2^nd ^internode (CD1 and CD2)

Two and one QTLs were associated with CD1 and CD2, respectively and were identified only in the C2-2 agro-ecology zone. These traits share common QTL regions on LG2 and the allele for thicker culms was contributed by Kaye Murri.

#### Peduncle length (PedL)

Eleven significant QTLs were identified on six linkage groups and five of the QTLs were associated with unlinked loci. The *R*^2 ^for PedL ranged from 0.11 to 0.35. At seven QTLs, *E. pilosa *(30-5) alleles increased PedL. Among these, two QTLs in LG10 and 21 were negatively associated with other traits (100sw and SB in LG10 and GY in LG21).

#### Panicle length (PanL)

Seven QTLs were identified for PanL, with a maximum *R*^2 ^of 0.22 and LOD = 4 for RZ588 in LG20. Kaye Murri alleles increased PanL in all QTLs, except for RZ251 (LG13) and RZ588 (LG20). Six PanL QTLs were associated with several yield-related traits.

#### Plant height (PH)

Four significant QTLs were identified with *R*^2 ^ranging from 0.13 to 0.26. Kaye Murri alleles at QTLs in LG2, 7, and 8 increased PH while the *E. pilosa *(30-5) allele increased PH at RZ588 (LG20). All PH QTLs were associated with QTLs for multiple yield-related traits.

#### Number of internodes (Ninter)

Three QTLs were associated with Ninter. The most significant QTL (LOD = 4.97, *R*^2 ^= 0.20) was on LG2 which was associated with PH.

#### 1^st ^and 2^nd ^internode length (Intet1 and Inter2)

Three and seven QTLs were identified for Inter1 and Inter2, respectively. These QTLs overlapped in LG13 where the *R*^2 ^was about 0.24, and longer internode length resulted from the *E. pilosa *(30-5)allele. The unlinked locus RZ961 was also associated with both of these traits.

#### Crown diameter (Dia)

Only one QTL, ISSR548a in LG8, was detected for Dia. This locus was associated with QTLs for nine different traits; PWt, PSWt, CulmL, PanL, PH, GY, SB, Lodg and Crush2 (Fig. 1). Most of these QTLs were unique to the DZBS location. Kaye Murri alleles increased crown diameter.

#### Crushing strength at the 2^nd ^internodes (Crush2)

Two QTLs were identified for Crush2. The traits of RPR and Crush were measured to evaluate the strength of culm in order to evaluate lodging resistance. However, QTLs for Crush2 (BCD1087a and ISSR548a) were not co-localized with QTLs for Lodg. RPR1, RPR2 and Crush1 did not show phenotypic variances among lines thus, QTL analyses were not available.

## Discussion

Single marker analysis (SMR) detects associations between individual markers and traits; therefore, it does not require a genetic map to be applied. In this study we used SMR for a preliminary test of significance of all polymorphic markers. For the loci that mapped into linkage groups [10], composite interval mapping (CIM) could be applied for detection and mapping of QTLs. Permutation tests were conducted to establish significant thresholds for CIM, reducing the chance of reporting false QTLs. In addition, multiple-trait analysis (MT-CIM) was used to analyze QTL over experiments, for detection of loci that consistently affected the phenotype across environments. The significant QTLs identified by all three analyses in common are presented herein (Table 3).

Tef improvement has relied mostly on mass selection from landraces for the development of new varieties. The grain yield of tef has risen from 3,425 to 4,599 kg/ha over 35 years of breeding [11]. The average rate of yield increase per year for the period of 1960 to 1995 was estimated at 27.16 kg/ha (0.79%), using linear regression of mean grain yield of cultivars on year of release. This gain is similar to rates reported for spring barley, oat and spring durum wheat in Ethiopia [11]. However, the national average grain yield of tef is still about 0.8 t/ha [1] and is not competitive with that of other major grain crops.

Grain yield was significantly correlated with all traits except PedL (Table 2). The associations of GY with HD, MD, PWt, PSWt, 100sw, SB, CulmL, CD1, CD2, PanL, PH, Inter1, Inter2 and Crush2 indicated that later maturing, taller, more vigorous, and larger plants resulted in more grain yield. Tefera et al. [7,8] showed most yield and yield related traits had high broad-sense heritability (*H*) in the population used in this study, and moderate to high *H *values were obtained in a population derived from an intra-specific cross. As expected, improvement of yield potential in tef has been associated with an increase of biomass yield and yield components. Among the 99 QTLs identified, 12 GY QTLs were detected in nine different linkage groups (Fig. 1). The map positions of the QTLs for yield related traits and SB on the same chromosomes overlapped, thus supporting the significant phenotypic correlations (*p *< 0.001) (Table 2).

Several chromosomal regions were associated with more than two traits indicating either linkage or pleiotropic effect. Clusters of QTLs (more than five QTLs) for various traits were identified on LG2, 3, 7, 8, 10, 13 and 20 (Fig. 1). Previous studies in cereal crops such as rice and wheat have also shown a clustering of agronomic QTLs [12-15]. The same chromosome region on LG21 was associated with positive and negative QTL alleles from *E. tef *for GY and PedL, respectively (Fig. 1), although the correlation between those two traits was non-significant (Table 2). The PedL QTL showed a similar relationship on LG10 with those of 100sw and SB which are yield related components. The association of two positive QTL effects in the same chromosomal region was reported for studies involving *O. rufipogon *in rice [13,16]. The allele of *O. rufipogon *had a beneficial effect where the increasing effect for grain yield was linked to decreasing effect for plant height [13]. However, in some cases beneficial QTLs from *O. rufipogon *were associated with undesirable QTLs. For example, a QTL increasing panicle length QTL was in the same region as a QTL increasing the proportion of broken grains [16]. Where associations of desirable and undesirable agronomic QTLs are in the same chromosomal regions, careful selection would be needed to avoid undesirable characteristics in the derived lines.

Epistasis is part of the genetic architecture of grain yield and other agronomic traits. Gene interaction has also been reported for a few phenotypic traits of tef [17-19] thus, it is not surprising to detect it for more complex quantitative characters in this study [20]. An analysis to identify the potential epistatic interactions between QTLs identified 20 marker loci resulting in 15 two-way interactions (Table 4). GY QTLs had five two-way interactions and TCD95 and lfm256 were actively involved in the epistasis. The most interesting interaction was between TCD95 on LG3, and TCD227a on LG8, for GY QTLs, because this was shown for SB QTL interaction as well (Fig. 1 and Table 4). In addition, QTLs on LG3 for GY and SB were detected in all three agro-ecology zones where agronomic traits were measured for this study. Likewise, the GY QTL (CNL53) on LG2 was detected across all three agro-ecologies and had significant interaction with TCD227a in LG8. Therefore, to improve grain yield, these three QTLs may need to be selected together.

Genotype and environment interaction could influence the ability to detect QTLs, even though tef displays versatile agro-ecological adoption with good resilience to both low and high moisture stress. Individual QTLs were not consistently detected across environments, and inconsistent QTL detection has been observed and attributed to QTL × environment interaction, which has been commonly observed in other grain yield QTL studies in cereal crops. Out of 12 GY QTLs, only two QTLs (LG2 and 3) were consistent across three agro-ecology zones. Three QTLs were detected in two agro-ecological zones: on LG7 (zones C2-1 and C3-3), LG8 (zones C2-2 and C3-3) and LG16 (zones C2-1 and C2-2). Even though, five GY QTLs were detected in multiple agro-ecology zones, there were no QTLs significant in all locations. The traits HD and MD as yield component traits are known to be sensitive to altitude because of day length. However, the HD and MD QTLs did not show discernible differences among different altitudes in this study. Assefa et al [21] demonstrated the diversity of yield related traits using 36 different germplasm populations collected from northern and central regions in Ethiopia corresponding to the same agro-ecology zones in this study. Regional differences in various traits of tef germplasm have been reported but altitude gradient regimes had no significant influence in affecting diversity levels in tef germplasm populations. Similar results were found in Ethiopian wheat, barley and sorghum germplasm [21].

Different soil types probably influenced QTL detection in this study. Two soil types were used in Debre Zeit: light soil (DZLS, Andosol, e04) and black soil (DZBS, Vertisol, e03 and e11). Plants were more vigorous and tall in the loamy Andosols, compared to the heavy textured Vertisol, even though the rainfall amount and temperature are the same for both soil types (Hailu Tefera, personal communication). The QTLs for PWt, PSWt, and Ninter were identified only at DZLS (e04), but the QTLs for 100sw, Lodg, PanL, and Inter2 were identified only at DZBS, 1999 (e03) (Table 3). Since those experiments were conducted at very similar conditions, it is likely that soil type was the major factor interacting with the QTLs. Teklu and Tefera [11] conducted a yield potential experiment in which 10 agronomic traits were examined for 11 tef varieties on two soil types. The most significant (*p *< 0.05) variety and soil type interactions were found for plant height and panicle length. Among four PH QTLs in this study, two were detected on LG7 (DZLS, e04) and LG8 (DZBS, e03) each. However, three QTLs for PanL were identified only in DZBS (e03), not in DZLS (Table 3). The environmentally sensitive QTLs for yield and yield components detected in this study clearly illustrate the importance of determining if QTLs by environment interactions are due to changes in magnitude or are crossover interactions before using MAS to select for QTLs. Identifying and selecting the proper allele at QTLs with crossover interactions requires careful evaluation in target environments. Inappropriate allele identification or selection could result in the indirect selection of QTL alleles with detrimental effects in some target environments.

Low grain yield of tef is partly due to the low basic productivity of currently available cultivars, together with susceptibility to lodging which has been the most serious agronomic problem. Lodging index showed positive and highly significant (*p *< 0.001) correlations with PSWt, 100sw, GY, SB and negative correlations with PedL thus, high yielding RILs tended to lodge (Table 2). Two of the Lodg QTLs, on LG8, were associated with PH, GY and yield related traits, and the other three QTLs were independent of yield related traits (Fig. 1). The positive correlation of lodging with yield and other important yield component traits indicates that improvement of lodging resistance in tef will be a challenging issue for a breeder. Of five Lodg QTLs, all alleles causing more lodging were from the tall, high yielding and more lodging resistant parent, Kaye Murri compared to *E. pilosa *(30-5) (lodging score 65.13 vs 81.50) (Table 1). This results from the unusual patterns of correlations of several traits differentiating the cultivated and wild parents of this cross. The weak or non-significant correlations of Lodg with CD1, CD2, PedL, PanL, PH, Inter1, RPR1, RPR2, Crush1, and Crush2 were counterintuitive. On the other hand, CulmL Ninter, and Inter2, were positively correlated while Dia was negatively correlated with Lodg as would be expected. The lack of significance of the negative correlation coefficients with RPR and Crush traits can be attributed to the small number of replicates and environments as well as the difficulty in measuring those traits. However, field observations of the wild and cultivated parent suggest that the very thin culms, small crown diameter, and weak straw of the wild parent, rather than plant height, are the traits contributing most to its lodging susceptibility. Several studies have found that QTLs for lodging and plant height are linked or located in the same chromosomal regions and could be used as indirect selection parameters for barley [22], rice [23], wheat [12], maize [24] and Italian ryegrass [25]. However, a reduction in plant height to improve lodging resistance may reduce the photosynthetic capacity of a canopy. In addition, the susceptibility to lodging differed among cultivars with similar plant height in wheat and rice [26,27]. Other factors such as stem cellulose or lignin content are related to stem rigidity [28] but were not measured in this study. One of the lignin biosynthesis genes, PAL (Phenylalanine ammonia-lyase from rice, X16099) co-localized with Lodg QTL in LG8 (Fig. 1) suggesting that it may be a candidate gene for this trait.

The development of inter-specific populations is one strategy to broaden the genetic diversity of cultivated crops and to identify QTLs associated with beneficial traits, such as yield, grain quality and disease resistance [29]. *E. pilosa *(30-5) alleles had an agronomically beneficial effect on 27 out of the 99 (27.3%) QTLs detected in the present study, including HD, PWt, PSWt, GY, SB, CD1, PedL, PanL, PH, Inter1, Inter, and Crush2. This proportion is similar to that reported by Septiningsih et al [30], where 33% of the alleles from the wild *O. rufipogon *presented favorable effects compared to *O. sativa *alleles. However, it is lower compared to the 53% reported by Thomson et al [15], with the same species. There were two QTLs identified on LG18 and LG20 with an increase in yield from the *E. pilosa *(30-5) alleles (Figure 1). The QTL on LG18 was not linked to any known undesirable QTLs and the *E. pilosa *(30-5) allele would be directly useful for developing breeding materials. However, the GY QTL interval (less than 10 cM) in LG20 was associated with a large increase in plant height, resulting in lodging. The GY QTL in LG20 may still be useful if the negative linkage can be broken or counteracted by other QTL reducing plant height. If markers can be successfully used to reduce linkage drag, the positive QTLs from *E. pilosa *(30-5) will be potentially useful for improving cultivated tef. Therefore, this study suggests that *E. pilosa *(30-5), and possibly other wild accessions, could be useful for diversifying the cultivated tef germplasm pool.

## Conclusion

The primary objective of this study was to determine the number and location of QTLs for important agronomic traits in tef. An inter-specific population was used to map 99 QTLs for 19 traits across 15 linkage groups. The interactions of genotypes and environments among QTLs were reported here to evaluate alleles for target breeding environments. The results of this QTL study are a first step towards the design of a marker-assisted selection program for tef improvement.

## Methods

### Mapping population construction

Two hundred recombinant inbred lines (RILs) derived from individual F2 plants of the cross *E. tef *cv. Kaye Murri and *E. pilosa *(30-5) were developed using single seed descent method at the Debre Zeit Agricultural Research Center (DZARC), Ethiopia. The cultivar Kaye Murri is characteristically later maturing, thick culmed, tall in stature, has a compact panicle structure, red lemma and white seed color. *E. pilosa *is early maturing, thin culmed, much shorter in stature, and has a loose panicle structure, extensive seed shattering, white lemma and dark red/brown seed color. These lines were phenotyped under field condition at three locations in Ethiopia in 1999. Of the 200 RILs, 181 lines survived across the three locations to generate phenotypic data. Moreover, some lines which showed mechanical contamination were further eliminated and 162 RILs were considered for a subsequent phenotyping in 2000. Ninety four RILs were used for construction of the linkage map of *E tef *cv. Kaye Murri × *E pilosa *(30-5) [10] and those RILs were used for QTL analyses reported in this study.

### Field trials

Twenty-two traits were evaluated at eight different locations in Ethiopia during the two-year period. In 1999, 200 RILs were planted in a randomized complete block design (RCBD) with four replicates at three locations (Akaki, Alemtena, and Debre Zeit Black Soil). Because of missing plots only 181 lines survived and were common across the three locations. In 2000, 162 lines were planted in a RCBD with two replicates at each of eight locations (Akaki, Alemtena, Debre Zeit Black Soil, Debre Zeit Light Soil, Denbi, Melkasa, Chefe and Holetta). The detailed information on field practices such as size of pots, pollination, fertilizer application etc. was described in Tefera et al. [7]. The eight locations were chosen based on their representation of the three major agro-ecosystems of tef in Ethiopia [31]. The humid zone (C1) in the Western regions of Ethiopia has a tef-growing period of more than 150 days and a growing season rainfall of more than 850 mm. The wet semi-arid (C2) in the Central parts of the country is subdivided into two minor areas, high altitude (C2-1) more than 1900 masl and low altitude (C2-2) with 1700–1900 masl. These areas receive a growing season rainfall of 450–850 mm and the growing period is between 100–150 days. The dry semi-arid or the Northern Rift Valley (C3) consists of three minor areas designated as high altitude (C3-1) more than 1900 masl, mid-altitude (C3-2) 1700–1900 masl, and low altitude (C3-3) less than 1700 masl. The eight locations for QTL analysis were as follows: i) C2-1; Akaki, Chefe, Holetta ii) C2-2; Debre Zeit Light Soil, Debre Zeit Black Soil, Denbi, and iii) C3-3; Alemtena, Melkassa.

### Trait evaluations

The RIL population was evaluated for 22 traits during the 1999 and 2000 growing seasons (Table 1). Ten plants per line were randomly selected at physiological maturity, and the following measurements were taken: (1) *Days to heading *(HD): number of days from planting to 50% of the plants in the plot showed panicle emergence. (2) *Days to maturity *(MD): number of days from planting to the day when 50% of the plants in the plot reached physiological maturity. (3 and 4) *Panicle weight *(PWt) and *Panicle seed weight *(PSWt): weight in grams of the panicle and the seeds harvested from the primary panicle, respectively. (5) *100 seed weight *(100sw): weight in milligrams of 100 seeds. (6 and 7) *Grain yield *(GY) and *Shoot biomass *(SB): total weight in grams of all the seed harvested from each plot and the remaining plant biomass after harvest, respectively. (8) *Lodging index *(Lodg): based on Caldicott and Nutall [32], which describes the lodging index as the sum of the product of each scale of lodging (0–5) and its percentage divided by five. (9) *Culm length *(CulmL): length in centimeters (cm) from the crown to the base of the panicle. (10 and11) *Culm Diameter at the 1^st ^Internode *(CD1) and *Culm Diameter at the 2^nd ^Internode *(CD2): width in cm at the middle of the first and second basal internode, respectively, using a caliper. (12) *Peduncle length *(PedL): length in cm last node and the bottom of the panicle. (13) *Panicle length *(PanL): in cm from the base of the panicle to the tip. (14) *Plant height *(PH): determined as the combined total of the culm length and panicle length. (15) *Number of internodes *(Ninter): the total number of internodes on the plant. (16 and 17) *1^st ^Internode Length *(Inter1) and *2^nd ^Internode length *(Inter2): measured as the length in cm of the culm section from the crown up to the base of the first node and second node, respectively. (18) *Crown diameter *(Dia): measured using a caliper and was determined as the width in cm around the middle portion of the crown. (19 and 20) *Rind Penetrometer 1^st ^*(RPR1) and *2^nd ^Internodes *(RPR2): measured as the force in kg required puncturing a 5 cm long section cut from the first and second internodes. Ten randomly chosen plants were collected at physiological maturity, and tests were performed on the main tiller. Two separate sections were cut 5 cm in length and 5 cm up from the base of each internode. (21 and 22) *Crushing strength 1^st ^*(Crush1) and *2^nd ^internodes *(Crush2): measured as the force in kg required crushing, to the point of bending, a cut stem section. The same 10 plants and stems used for the puncture resistance tests were used for this measurement. Stem sections were cut 10 cm up from the base of each internode and 5 cm in length. The sections were dried at air temperature for 4 weeks before measurement. In total, 22 traits were measured in the 11 different experiments and classified as yield and yield related traits or morphological and plant related traits. The traits recorded in each experiment and the trait designators used are given in Table 1.

### Statistical analyses

The genetic linkage map for the 94 RILs reported by Yu et al. [10] was used in this study. Briefly, 142 molecular markers produced 192 segregating loci; among those, 156 loci linked into 21 groups and 36 loci were unlinked. The map was constructed using restricted fragment length polymorphism (RFLP), simple sequence repeats derived from expressed sequence tags (EST-SSR), single nucleotide polymorphism/insertion and deletion (SNP/INDEL), intron fragment length polymorphism (IFLP), targeted region amplification polymorphism (TRAP) and inter-simple sequence repeat amplification (ISSR). The map covered 2,081.5 cM with a mean marker interval of 12.3 cM.

Phenotypic data were analyzed in SAS System V.8 [33]. The normal distribution of phenotypic data was verified using Shapiro-Wilk test at *α *= 0.01, and in some cases required transformation to log or square-root. Analysis of variance was done for each experiment, and line means were used for QTL analysis. Pearson's correlation coefficient was computed among phenotypic traits. QTL analyses were implemented in QTL Cartographer Version 2.5 [34]. First, data were analyzed to identify markers associated with variation for each trait using single marker analysis (SMR) using all linked and unlinked loci at a statistical threshold of *p *< 0.01. Second, trait data were analyzed by composite interval mapping (CIM) [35], using a reduced set of unlinked marker loci containing significant loci detected by SMR analysis. The parameter settings for CIM were model 6, forward and backward stepwise regression with threshold of *p *< 0.01 to select cofactors, window size 10 cM and 2 cM walking speed along chromosomes. QTLs were verified by LOD sores compared to an empirical genome-wide significance threshold calculated from 1,000 permutations for *p *< 0.01 to control type-I error. QTL position, LOD score, coefficients of determination (*R*^2^), and additive effect were estimated by CIM for each QTL. Third, multiple-trait analysis method (MT-CIM) was used to jointly analyze QTL over experiments using the value of a trait in different experiment as a correlated trait [36]. The analysis through MT-CIM was performed using the parameter settings above, and LOD = 3.5 for declaring QTLs. Fourth, the Epistat program [37] was used to identify and evaluate pairs of loci whose combined effects can not be explained by independent and additive action using maximum likelihood together with Monte Carlo simulations.

## Authors' contributions

**JY **participated in data analyses and drafted manuscript. **EG **conducted the field studies in 2000 in cooperation with the tef research staff in Ethiopia. **FB **helped in analyses of data and consulted statistical analyses. **HT **produced the cross, developed the RILs and coordinated the field studies. **MES **conceived of the study, and participated in the design of the study, data analyses and manuscript preparation. All authors read and approved the final manuscript.
